# Telling Apart Motor Noise and Exploratory Behavior, in Early Development

**DOI:** 10.3389/fpsyg.2018.01939

**Published:** 2018-10-12

**Authors:** Teodora Gliga

**Affiliations:** ^1^School of Psychology, University of East Anglia, Norwich, United Kingdom; ^2^Centre for Brain and Cognitive Development, Birkbeck, University of London, London, United Kingdom

**Keywords:** variability, infants, reaching, vocal behavior, exploration

## Abstract

Infants’ minutes long babbling bouts or repetitive reaching for or mouthing of whatever they can get their hands on gives very much the impression of active exploration, a building block for early learning. But how can we tell apart active exploration from the activity of an immature motor system, attempting but failing to achieve goal directed behavior? I will focus here on evidence that infants increase motor activity and variability when faced with opportunities to gather new information (about their own bodies or the world) and propose this as a guiding principle for separating variability generated for exploration from noise. I will discuss mechanisms generating movement variability, and suggests that, in the various forms it takes, from deliberate hypothesis testing to increasing environmental variability, it could be exploited for learning. However, understanding how variability in motor acts contributes to early learning will require more in-depth investigations of both the nature of and the contextual modulation of this variability.

## Introduction

Watching infants move about, interact with objects or attempting to communicate, one cannot but observe the great variability of their motor acts. A 7 months olds’ repeated banging of an object on a hard surface takes many trajectories. From one movement to the next, she might be grasping the object differently, as if exploring both the motor affordances of the object and her own motor abilities. Exploratory behavior, or the focused investigation as children get familiar with new environments (Hughes, 1978; Zosh et al., 2018) was proposed as a driving force for learning. However, deciding whether variability in motor acts is actively produced to serve learning, is not straightforward. Gibson, in her 1988 monograph, had already noted the difficulty with interpreting early motor acts. While suggesting that “The active obtaining of information that results from the spontaneous actions of the infant is a kind of learning,”" she also raised the question of whether “this activity is in any way controlled by the infant,” rather than “compulsory response to stimulation (Gibson, 1988).” This is further complicated by the fact that variability in motor behavior has often been described as the manifestation of an immature system, which is attempting but failing to achieve goal directed behavior (e.g., Yan et al., 2000). In the adult skill acquisition literature, as well, movement variability is an index of error or noise in sensory-motor systems, something the organism strives to eliminate as a new skill is acquired (Harris and Wolpert, 1998; Todorov and Jordan, 2002). Understanding under which conditions variability reflects exploration rather than noise is critical for both those interested in identifying and intervening in atypical development and for those invested more generally in creating environments that offer opportunities for learning. This review aims to create a framework for the study of early motor acts as exploratory behavior.

I will start by reviewing evidence suggesting that increased motor variability is not always a manifestation of an impaired or immature motor system since (1) variability sometimes increases in development and (2) decreased and not increased variability was documented in certain developmental disorders. I suggest (joining others, e.g., Thelen and Smith, 1994) that variability is upregulated when an organism faces new learning opportunities. I will propose this as a defining principle that sets apart variability as exploration from variability that is simply noise. This will be supported by evidence for (3) an increase in the amount and variability of motor output with information availability, in experimental situations, and for (4) direct associations between variability in motor activity and learning outcomes. I will then move on to discussing the mechanisms that might support the upregulation of motor variability and those linking variability to learning. Given the aim of this review is to illustrate a general principle, evidence will be brought from a variety of motor acts: reaching, locomotion or vocal behavior, and from a variety of species. Both variability that supports exploration of an organism’s motor abilities and of the surrounding environment will be considered. Finally, I will ask the question of which of these mechanisms might be at play in early human development.

### Measuring Variability

**FIGURE 1:**
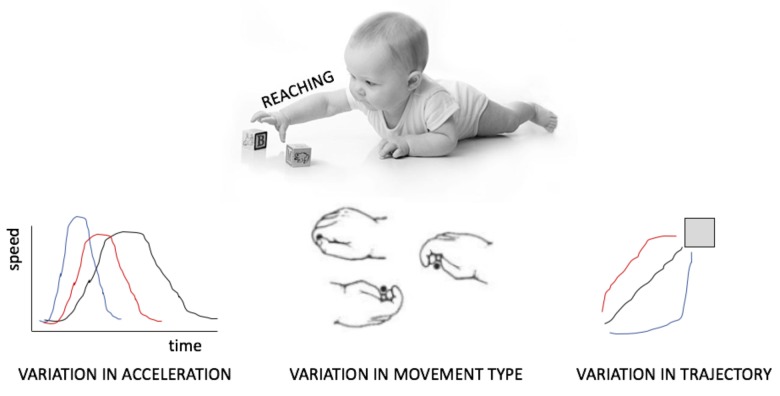
Different manifestations of variability in motor output, here exemplified for reaching and grasping.

This review does not draw on a rich literature. Although many studies have characterized the amount of movement or the types of movements infants produce, few have focussed on the manner in which acts are realized, as for example on the variability in acceleration, trajectory or in the combination of articulators (**Figure 1**). Even fewer have investigated this variability as exploratory behavior. Those who have done this, have sometimes distinguished between *variability*, calculated, for example, as the sum of the variance at each point in the path of a reaching hand (Wu et al., 2014) and *complexity*, which takes into account the temporal dimension of this variation, such as the amount of repetition of the same type of sway movement when standing (Dusing et al., 2013). It remains unknown which of these measures better captures variation targeted at exploration. By inquiring putative neural mechanism generating variability, this review hopes to offer guiding methodological and theoretical principles that can fuel a new avenue of investigation.

## Evidence That Variable Behavior Reflects Exploration

### Increased Variability at Key Points in Development

**FIGURE 2:**
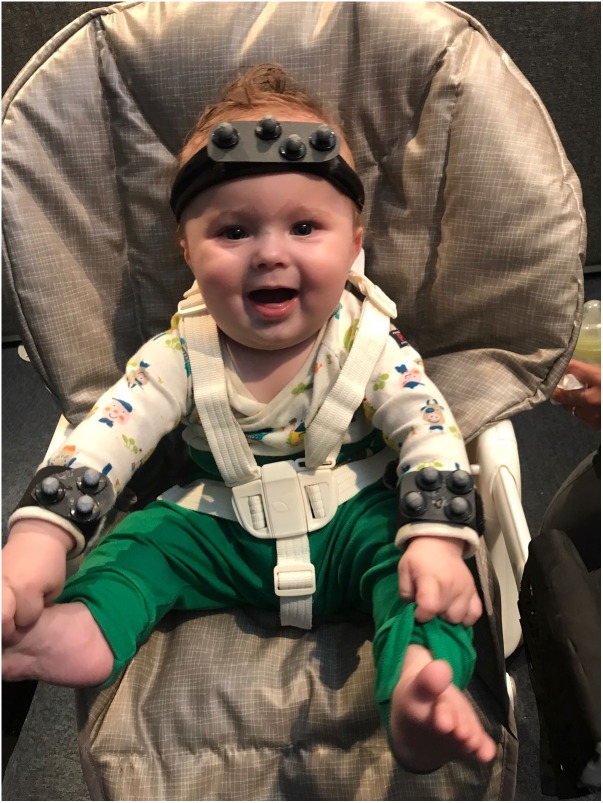
Motion tracking systems allow for precise tracking of infant’s limb or head movement. The position of light reflecting spheres attached to the infant body is triangulated with the help of a system of surrounding cameras. This system is light and does not interfere with infant’s movements.

If variability is a nuisance then we’d expect development to always proceed from more variable to less variable behavior. On the contrary, observing increasing variability, at certain moments in development, might point to it having a functional role. Dynamic systems accounts of development have already highlighted the need for transitions between stability and variability, whenever new skills emerge (Thelen and Smith, 1994). Increased variability has been observed at various points in development. Motor activity starts early in fetal development. Fetal movements are varied and structured; rodents, for example, exhibit coordinated motor patterns antecedent to postnatal locomotion, suckling, maternal–infant communication and grooming behavior (Robinson and Smotherman, 1992). This activity decreases toward 40 weeks after gestation, whether the pup is born at term or pre-term, suggesting that this decrease does not reflect space limitation toward the end of the pregnancy but a pre-programmed pattern of up and down-regulating variability (Robinson and Smotherman, 1992). Indeed, after birth, although the newborn must cope with the restraints of gravity, there is an increase in the variability of movements. In human infants, we see the emergence of writhing general movements (Prechtl, 1993). These variable sequences of arm, leg, neck and trunk movements, with often slight changes in direction of the movement “make the movements fluent and elegant and create the impression of complexity and variability” (Prechtl, 1993). An increase in combinatorial variability, in terms of a decrease in the locking of movement of different limbs is also observed from 6 to 18 weeks (Piek et al., 2002). Despite the repeated suggestion that an increase in variability reflects an active process of exploration, allowing the selection of most efficient movement strategies (Edelman, 1987; Stulp and Oudeyer, 2018), this process of increasing variability followed by selection has not yet been captured, in development. This limitation is most certainly methodological, since new skills appear at different points in time in different infants (e.g., infants may start crawling anywhere from 6 to 12 months, and some skip this locomotive stage all together), and capturing these transition points would require frequent sampling before and after the new skill emerges. New wearable technologies (see **Figure 2**), might make this research easier to carry out. Alternatively, one can attempt to train new skills in the lab (see further on).

### Variability in Atypical Development

Another piece of evidence in support of the idea that variability promotes development and learning comes from observations of decreased rather than an increased variability of motor outputs in many pathologies of movement (e.g., Parkinson’s Disease - Freund and Hefter, 1993; stuttering-Grosjean et al., 1997) but also more generally whenever development is compromised. This is the case in infants with documented brain damage, who display monotonous and more stereotypical movements, less fluent and lacking complexity (Newell et al., 1993; Prechtl, 1993). Cerebral palsy has also been linked with decreased movement variability in the first few months of life (e.g., Prechtl, 1997). Vaal et al. (2002) showed that 18 and 26 weeks old infants with periventricular leukomalacia had tighter intra-limb locking during spontaneous kicking, compared with infants with no evidence of brain damage. 9-month-old high-risk preterm infants engaged in less fingering, rotation or transfer of objects and a summary exploration score predicted cognitive functioning at 24 months (Ruff, 1984). Preterm born infants showed decreased variability in reaching movements, both when producing distal (e.g., reaching with both one or two hands) and proximal adjustments (e.g., various hand openings; Soares et al., 2014) and lower levels of exploratory movements of toys (Soares et al., 2012; Guimarães et al., 2013). Between 12 and 18 months of age, when infants start standing up unsupported, variability in the execution of this motor act is the norm; the rotation of the foot, the degree of knee flexion and hip abduction or the foot leading the movement vary from one standing up to the next (Towen, 1993). Atypically developing infants make fewer attempts to stand up but the most striking difference is in the decreased variability of the gestures (Towen, 1993).

However, some pathologies are associated with increased variability. In Tourette’s there is poor motor learning but increased variability (e.g., Draper et al., 2015). Hyperkinesia and extreme clumsiness are often observed in development and characterized by increased variability (Towen, 1993). Towen (1993) noted that this apparent discordant evidence probably reflects poor understanding of the mechanisms generating and making use of variability. He advanced the idea that in some pathologies, it may not be the mechanisms generating variability but the selective process (of optimal motor strategies), that is impaired. Alternatively, it may be the nature of the variability, reflecting decreased exploration or increased noise, that differs between pathologies. Investigating whether variability increases, or fails to increase, in learning contexts, may help tease apart between these hypotheses.

### Variability Increases With Information Availability

Since exploratory behavior is behavior targeting information acquisition, an increase in variability when new information is available is a key indicator of variability as an index of exploration. It was indeed observed that, when infants are engaged in reaching training regimes they initially produce distal adjustments that increase in variability (Soares et al., 2013). Across a number of studies, introducing infants to an object with a new property increased object-directed movement and the variability of movement types. Steele and Pederson (1977) observed that 6-month-old infants increased their touching and looking behavior when introduced to an object that differed in temperature from previous ones, but no change in behavior occurred when the object changed color. 9- to 12-month-olds engaged in more banging when exploring objects that had a new weight and more rotating and transferring when exploring objects that had a new shape (Ruff, 1984). When given an object with a new texture newborns increased the frequency of their hand pressure movements (Molina and Jouen, 2004). In another study, information content modified infants mouthing of artificial nipples – more variable movements (and less sucking *per se*) was measured in newborns when they experienced a new nipple texture (Rochat, 1983). Rochat notes that this activity could not have been reflexive, since it was modulated in character and varied according to context.

Later in development, it was observed that vocal articulators increase in movement variability following cochlear implantation. The stability of movement trajectories for correctly produced speech was compared pre- and post-implantation, in a 7-year-old child (Goffman et al., 2002). Pre-implantation, the participant had slightly higher movement variability than age-matched controls. Two and four months after implantation, variability increased further but by 6 months post-implantation, this child produced speech movements of a similar stability as the controls. The authors comment that variable movements of correctly articulated speech ‘may reflect a system that is being modified in response to new auditory input provided by the cochlear implant’ (p. 892).

Although these studies are compatible with the idea that variation is upregulated to help learning, it still remains possible that an increase in variability in learning situations simply reflects failed attempts to achieve a new goal rather than a process of exploration. Showing that increasing variability actually leads to knowledge accumulation, provides the strongest evidence for its adaptive role in development or skill acquisition. This type of evidence remains scarce.

### Variability Leads to Learning

One of the most compelling studies leading variability in motor activity and learning, had adult participants learn a new motor routine. Wu et al. (2014) measured variability of arm trajectories before and during a motor learning task in which participants had to draw subtly curved shapes, with fast arm movements. Variability during a baseline period (in which no feed-back was given for tracing a model curve), was positively correlated with how close to the target curve participants got in the training phase. It was variability in a task-relevant dimension that best predicted learning. In another study, Byun et al. (2014) assessed the role of motor exploration for vocal learning and found that children enrolled in an ultrasound biofeedback intervention for /r/ mis-articulation only made progress when they were allowed to try out a variety of tongue shapes for /r/, rather than being set a specific shape by the therapist. More recently, Lee et al. (2018), showed worse learning of a new motor skill in children than adults. Participants had to use their upper body movements to control a cursor on a screen. The authors explained these findings based on children’s limited exploration of their movement repertoire. Exploration was quantified here as the ratio between the 2 principal components that explained most variance in movement. This metric was considered to better capture exploration of the 2 dimensions of the screen than variation within each dimension.

No study yet has shown that progress in a particular learning task is improved in infants that manifest increased variability in behavior (e.g., better discrimination of weight in infants that had manifested most variability in banging objects or acquiring faster reaching in those infants that started off with higher reach variability). A recent study took a different approach to demonstrating this relationship, by simulating learning of the ability to play football in conditions of variable or non-variable walking practice. Rather than using human infants, Ossmy et al., 2018 used robots. As predicted, training that varied in path shape, step direction and number of steps helped teams win “RoboCup” tournaments. Although this first study did not investigate the role played by different types of variability, this approach clearly has the potential to delve deeper in understanding the mechanisms linking variability to learning.

## The Mechanisms Driving Variability in Motor Acts and Its Contributions to Learning

A mechanistic understanding of how variability is actively generated may also help us identify it and understand how it supports learning. One strategy is to look at where in the nervous system variability originates. In a recent review, Dhawale et al. (2017) differentiate between *planned noise*, variability generated in the central nervous system and *execution noise*, variability resulting from the randomness of biological processes such as spike generation and propagation, synaptic transmission, muscle protein changes; however, execution noise may originate both in the central and the peripheral nervous system. Thus, variation in cortical activity does not necessarily reflect actively generated variability.

However, specific mechanisms have been suggested to generate variable behavior that may point to specific manifestations of variability. In its highest-level form, planned variability may reflect deliberate *hypothesis testing*. Children figuring out how to activate a hidden mechanism with the help of wooden blocks try various combinations of blocks and often verbalize the hypothesis they are testing (Gopnik et al., 2001). This process of hypothesis or theory testing is seen by some as critical for advancing learning, especially for generalizing knowledge beyond the particulars being experience at a moment in time (e.g., see Annette Karmiloff-Smith’s, “If you want to get ahead, get a theory”; Karmiloff-Smith and Inhelder, 1974). Discrete instantiation of each hypothesis, especially when accompanied by verbal explanations, clearly identifies this process as exploration. How exactly hypotheses are generated remains largely unknown; in their study of balancing objects, Karmiloff-Smith and Inhelder (1974) observed that past experience heavily influences which hypothesis children will try out (try balancing in the middle if that worked before), and formulating a new theory – and therefore trying out a new balancing point – did not necessarily emerge from encountering counter-examples of the former theory, but from a process of insight, difficult to capture from children’s behavior.

There are however, cases in which an individual does not have enough background knowledge to formulate explicit theories. To take a simple example, we might know where alternative sources of food could be if we don’t find any in our fridge (try corner shop); but while in a forest and hungry, we might not even recognize what food looks like. Adopting quasi stochastic behavior, e.g., sampling anything that looks vaguely edible, might be our best bet in these situations. This trial-end-error approach is critical for reinforcement learning. Rats faced with an unpredictable competitor for food, whose actions they try to counteract but fail, adopt a random pattern of choices between two food sources (Tervo et al., 2014). Another example comes from song learning, in zebra finches. Young males produce song syllables with a normal distribution of pitch values (Tumer and Brainard, 2007). Tumer and Brainard (2007) showed that by negatively reinforcing the upper end of a normal distribution of pitches through the contingent presentation of white noise, the pitch of a particular syllable in the song can be shifted. Interesting, the shift resulted in a distribution with a new mean, but which maintained the same degree of variability around the mean. Thus, this variability is actively maintained to enable the learning of new songs through reinforcement of particular ranges in the distribution, just like genetic variability is generated for natural selection to occur. Arm reach angles of adult human participants learning a new motor task are also initially normally distributed around an optimal value (Pekny et al., 2015). I will call this *learning expectant variability*.

Despite the seemingly stochastic nature of this variability, some have argued that it is not simply reflecting execution noise, but is actively produced at the motor planning stage. Churchland et al., 2006 showed that about half of the variability in reach speed (in monkeys) originates in the pre-motor and motor cortex. The neural structures and physiological mechanisms through which pitch variability is produced in the finches’ brain are also well characterized (Budzillo et al., 2017). However, as stated before, cortical origin does not necessarily imply active modulation of variability. The strongest evidence in support of the active generation of variability in these cases comes from the fact that variability is contextually modulated and increases in situations conducive to learning. For example, the song of young male zebra finches increases in spectral variability when they sing in isolation, compared to when singing to a female (Kao and Brainard, 2006; Budzillo et al., 2017). Thus, males take advantage of solitary moments to explore vocal productions, in view of improving their song. In rats, it is the presence of a novel, uncertain environment that activates noradrenergic input from the locus coeruleus into the anterior cingulate cortex (ACC). This suppresses ACC activity (i.e., responsible for accessing previous world models), leading to an upregulation of motor variability (Tervo et al., 2014).

However, some variability in motor acts is simply noise. Could learning take advantage of this type variability as well? We can see why this is difficult by taking an example from learning sensory-motor contingencies. In a system with high execution noise, erroneous contingencies between intended motor plans and the actual (incorrect) motor output may be created. However, studies that have used passively generated variability suggest that sensory feed-back is sufficient for reinforcement learning to occur. For example, in Bernardi et al. (2015) adult participants learned a new motor contingency after only having been given passive exposure to a variety of trajectories to a particular target, some of which were reinforced as successful hits. Passive exposure was achieved by moving participant’s limbs using a robot arm and resulted in the same learning success as active training. Thus, even in the absence of motor plan, participants could discover successful motor sequences simply based on the sensory feed-back they received from their limbs. However, in this case, recovery of the motor plan was possible by the existence of known sensory-motor contingencies. Participants were adults who had a life time of experience with arm movements and therefore a fairly good idea of which of which motor plans could lead to the particular sensory feed-back. These assumptions will not hold at some point in infancy.

## Which of These Mechanisms Could Generate Exploratory Variability in Infancy?

Where might infant variable motor outputs be, on the continuum between hypothesis testing and sensory-motor noise (see **Table 1**)? Gibson (1988) suggested that infant exploratory activity “continues as play through the preschool years and as deliberate learning later in life,” and this possibly reflects the view of many others. However, even for a gesture as simple as reaching, we have little evidence for developmental continuity between the mechanisms driving the various paths arm movements when a 4-month-old reaches for an object, when a 12-month-old tries to activate a new mechanical toy, or when, a year later, she figures out how to build a tower of blocks. It is highly likely that the balance between noise and deliberate exploration, as sources of variability, shifts during development.

**Table 1:** Potential sources of variable behavior in early motor output.

Sources of variability	Linked to learning	Present during infancy
Hypothesis testing	Yes	Stahl and Feigenson, 2015
Learning expectant variability	Yes	Soares et al., 2014
Environmental variability	yes	Fagan and Iverson, 2007
Sensory-motor noise	unclear	Certainly

### Hypothesis Testing

Many have been captivated by the metaphor of infants as little scientists (see Alison Gopnik’s “Scientist in the crib”), and indeed some early object exploration descriptions do seem compatible with primitive hypothesis testing. Infant’s using different action patterns when reacting to changes in object properties (e.g., Steele and Pederson, 1977; Ruff, 1984) could reflect deliberate testing of a “perceptual” hypothesis, e.g., banging might reflect infants’ deliberate testing object weight and fingering, the optimal way of testing an object’s temperature. These behaviors are very similar to the exploratory procedures used by adults when having to discriminate objects based on various properties (Lederman and Klatzky, 1993). However, these behaviors need not reflect infants apriori appreciation that banging is a better way of learning about weight than about temperature. It may simply be that the unexpected change in temperature triggers exploratory behavior, just as unexpected environmental changes increase randomness in motor choices in rats (e.g., Tervo et al., 2014). An increase in a variety of object directed actions (banging, fingering, mouthing) would eventually allow infants to discover that some of these actions bring about more information than others – e.g., that a new object’s temperature is better perceived when fingering it. Fingering would therefore be gradually selected over other behaviors, in the process of infants interacting with objects but may not initially be stored in long term memory as an explicit strategy to use for learning about temperature. Younger infants may have to make this discovery at each encounter of a temperature change. Rather than hypothesis testing, this would, at least initially, variable movements in object exploration may initially reflect learning expectant variability.

Hypothesis testing was directly investigated in a recent study by Stahl and Feigenson, 2015. Here, 12-month-olds manipulated objects differently following solidity vs. support violations – they banged objects that had passed through walls, but dropped objects that had not obeyed gravity. In this case, the objects themselves did not give away any cues about their properties, which means infants must have apriori chosen which actions were best suited to test their previous observations. In another study, Needham and Baillargeon (2000) observed an intriguing association between the percentage of time 3.5-month-old infants looked at or mouthed objects they were holding and their ability to visually parse objects based on their surface features. While this might simply reflect that motorically advanced infants also have better visual processing skills, an alternative interpretation is that object manipulation, which involves breaking contact between objects, had helped infants formulate hypothesis about object structure, for example the hypothesis that discontinuity in surface features will result in objects being easily taken apart.

Is talking about hypothesis testing, in the above cases, too rich of an interpretation of infant’s behavior? In its simplest form, the hypotheses infants test involve acting on the world and expecting a particular outcome (e.g., when I bang this object, I will perceive its weight). But is it possible to demonstrate that infants build up specific expectations during exploration? In an EEG study in which infants could build specific expectation about learning either object functions or labels, theta-band activity was measured over frontal areas in anticipation of object functions, but temporal theta activity was measured when labels were expected (Begus et al., 2016). Frontal theta band activity was measured also while infants explored objects (Begus et al., 2015). These neural correlates of information expectation offer an opportunity to investigate the earlier forms on hypothesis testing driving infant object exploration.

### Learning Expectant Variability

Is there evidence that infants produce the type of learning expectant variability that supports reinforcement learning? The increasing variability in reaching behavior during the first year of life, may be a good candidate for this mechanism at play in infancy (Thelen, 1979; Prechtl, 1993). Infants given reaching and grasping practice, which includes reinforcement of successful reaches, increased the frequency of this behavior (Soares et al., 2013). Interestingly, training only increased grasping success in infants born at term (Soares et al., 2014), i.e., in those infants that showed higher variability in grasping behavior already before the intervention. This suggests that increased variability may give term infants more opportunities to discover optimal reaching strategies. However, only one published study reports on an attempt to directly reinforce a subset of the spatial positions that 5-month-old infants’ hands took during reaching (Darcheville et al., 2004), a manipulation similar to the reinforcement of particular pitches in zebra finches’ song. In this study, the arrival of infant’s hand within particular spatial positions was automatically detected and generated a recording of mother’s voice. This manipulation increased reaching behavior; we do not know, however, whether this was accompanied by an increase in reaching using the reinforced trajectory. Selective reinforcement of either consonants and vowels (through smiling, vocal responses and touch) works to increase infants’ production of these phoneme classes (Routh, 1969). There is some evidence that mothers themselves selectively reinforce infant vocalizations, as for example imitating infant consonant production more than vowel productions (Gros-Louis et al., 2006). Again, evidence for reinforcement of vocal behavior also falls short of telling us whether learning takes advantage of the increased variability in infants’ vocal productions, for example.

### Environmental Variability

One obvious source of variability in behavior is the environment itself. For example, when reaching for an object, another object might block her way and change the reaching trajectory; reaching might change an infant’s center of gravity and this in turn could affect the trajectory her arm takes toward an object. Reinforcement learning is central to computational models of reaching (Caligiore et al., 2014) and vocal development (Moulin-Frier and Oudeyer, 2012) and these models critically depend on an initial pool of variable behavior. To model reaching development, Caligiore et al. (2014) used what they call exploratory noise, i.e., random perturbations in the motor output, to which muscular noise is added. Interestingly, these authors suggest that much of the exploratory noise is not actually planned by the child, but is a consequence of the child interacting in with her (unpredictable) environment. However, is there evidence that the child herself could generate environmental variability with the aim of exploring their body or the environment?

Fagan and Iverson (2007) suggested that one of the functions served by object mouthing, during early play, is to add variability to vocal output – creating some kind of lucky accidents. These researchers went on to show that a larger variety of glottal and sub-glottal sounds were produced when infants were vocalizing while mouthing objects. However, none of the sounds produced were new, in the sense that they were well in the repertoire of an infant that age. Mechanistically, this type of variability is not different from variability produced by noisy motor outputs, since the child is not in control of it, i.e., not in possession of the motor plans that yielded the final behavior. *A priori*, these motor plans could still be retrieved by making use of the sensory output of these actions and mapping them back on their motor plan(s). Of course, had the sound produced been a new sound, a corresponding motor plan would not exist. This strategy of increasing vocal variability is therefore unlikely to a driver of phonological development. The best a learner can do, if a new sound results from them mouthing objects, is to access the nearest motor plan available, the motor plan corresponding to the closest sound in their repertoire. Given young infants poor memory, sifting through these motor plans should occur fast enough, before she forgets the sound she wanted to re-enact. The solution to that is one other feature of early exploratory activity. In addition to being highly variable, exploratory behavior is also highly repetitive, in the sense that the same motor act may be activated many times in a row.

Repetitive patterns of behavior are present in both limb and vocal movement, and in higher frequency at particular time points in development. Cyclical grasping is elicited in 3-day-old infants when they are handed objects with new textures (Molina and Jouen, 2004). Repetitive actions with objects are present at high frequency during infancy, being ubiquitous at 12 months (Fyfield, 2014). Repetitions per vocalization increase and peak around 9.5 months (Fagan, 2009) but decline with word production (only 18% of first words contain 2 reduplicated syllables Vihman, 1996). With increase motor control, infants could actually produce more reduplication, but they do not. Thus, the amount of reduplication does not reflect competence, but seems to serve a particular function during particular windows of development. I suggest here that this type of repetitive behavior may help infants recover the correct sensory-motor mappings. A detailed analysis of reduplicated behavior will reveal that repetitions are not identical. Although the same motor plan is activated, variability in outcome is the result of added execution noise. Given this noise is normally distributed, with the most common outcome at noise zero, this should allow the mapping of the motor plan onto the correct output. The role reduplication has in learning new sensory-motor mappings is suggested by the fact that reduplication decreases in the absence of sensory feedback. Deaf infants show delayed or absent reduplication (Oller and Eilers, 1988; Koopmans-van Beinum et al., 2001). Reduplication does appear in vocal production weeks after cochlear implants and, interestingly, precedes an increase in the quality of the consonant vowel vocalizations themselves (Fagan, 2015). However, strong evidence in support of this hypothesis will come from precise measurements of the motor parameters of repetitive motor acts. This has now become possible thanks to motion tracking technology (**Figure 2**).

## Conclusion

We set to answer the question of whether the variability characteristic of infants motor acts is actively generated, rather than being the signature of an immature motor system. Evidence for contextual modulation of motor variability, especially evidence that variability increases with information availability, and a better understanding of the neural sources of variability, suggests that, even early in development, variability might be upregulated in support of learning. However, strong support for this hypothesis still awaits a better characterisation of infant motor variability *per se*, in the same way in which it has been characterized in bird vocal learning or adult motor skill acquisition. A better characterization of how variability in motor outputs is modulated in learning contexts will allow us to understand to what extent they reflect hypothesis testing, learning expectant variability, or merely infants actively creating lucky accidents.

## Author Contributions

TG has written this review paper.

## Conflict of Interest Statement

The author declares that the research was conducted in the absence of any commercial or financial relationships that could be construed as a potential conflict of interest.
